# On recovery: re-directing the concept by differentiation of its meanings

**DOI:** 10.1007/s11019-021-10014-7

**Published:** 2021-04-03

**Authors:** Yael Friedman

**Affiliations:** Centre for Philosophy and the Sciences (CPS), Department of Philosophy, Classics, History of Art and Ideas, University of Oslo, Oslo, Norway

**Keywords:** Recovery, Medical epistemology, Phenomenology, Epistemic injustice, Habilitation, Medical holism

## Abstract

Recovery is a commonly used concept in both professional and everyday contexts. Yet despite its extensive use, it has not drawn much philosophical attention. In this paper, I question the common understanding of recovery, show how the concept is inadequate, and introduce new and much needed terminology. I argue that recovery glosses over important distinctions and even misrepresents the process of moving away from malady as "going back" to a former state of health. It does not invite important nuances needed to distinguish between biomedical, phenomenological, and social perspectives. In addition, I claim that there are many conditions where we are making use of the concept of recovery, although the person recovered from the condition in question, has not regained the same degree of soundness. I show how the concept of recovery leads to conceptual discrepancies that can result in worsening patients' conditions. To gain a fuller understanding, I propose to rethink the direction of the process in question. I define the process of moving away from malady as a move forward towards a new state of soundness. I also suggest three terms, corresponding to different perspectives, to describe this movement forward: 'curing' (biomedical perspective), 'healing' (first-person perspective), and 'habilitating' (social perspective). This new terminology provides a more nuanced understanding of the states of both malady and soundness and an attentiveness as to how they differ and relate.

## Introduction

Medicine and health care professionals, health policymakers, and English speakers use the term «recovery» as a general concept referring to the process of regaining health, well-being, or a lost ability, including the ability to work and perform in society.^1^ The etymology of the word 'recovery' is to return or come back (Merriam-Webster's-Dictionary n.d.) to the stage prior to becoming diseased, ill, or sick or before the injury occurred.^2^ This means that recovery is achieved when a patient who experienced a malady comes back to his or her former sound state.

Although the concept of recovery is widely used in both daily life and professional contexts, it has not drawn much philosophical attention. Philosophers of medicine have shown great zeal in defining related concepts such as health, illness, and disease while using recovery as a general mediator between these conditions. As will become apparent through the course of this paper, a closer look at the concept of recovery shows that it represents many of these states of affairs inaccurately. Moreover, the use of this concept is not in line with current literature in philosophy of medicine, which is attentive to the distinction between the various agents involved in a medical situation, see for instance (Broadbent 2019; Carel 2014, 2016; Hofmann 2002, 2016; Sholl 2020; Svenaeus 2019). The concept of recovery as it is currently used lacks important nuances and creates conceptual discrepancies regarding individuals’ conditions. These gaps can lead to ‘epistemic injustice’ (Fricker 2007) by causing us to privilege some agents' perspectives on recovery while rejecting those of others as epistemically relevant, although they are in principle no less pertinent. Epistemic injustice can worsen people's conditions both directly and indirectly. It can do so directly, for example, by recognizing them as no longer eligible or in need of treatment when in fact they are. And it can do so indirectly by no longer providing them with social benefits they should have been eligible for or by tagging them as incapable, although they have recovered. Thus, there is a need to make the concept of recovery more precise and applicable to individuals’ circumstances.

In this article, I will criticize the use of the concept of recovery as being too *general* since it incorporates *different ideas* of recovery without differentiating between them, and as being *inaccurate* since we use the concept of recovery extensively when patients *have not come back* to their former sound state. First, I will analyze the concept of recovery by discussing four conditions from which one can be considered as 'recovered'—*disease*, *illness*, *sickness*, and *injury*—and the extent of their overlap with each other. Second, I will show that we also use the concept of recovery when the new sound state of a patient improves or worsens from the state before the malady occurred, as well as when one cannot evaluate the relation between the two stages, such as in the case of immunity of COVID-19 recoverers. In the final section of the article, I will suggest alternative terminologies that overcome the problems with the concept of recovery. I will propose using three different terms to indicate the patient's improvement from different perspectives and understanding the process of moving away from the malady as a process that aims forward (and not backwards)—from the malady stage to a new soundness stage. This view allows for a dialogue about different conditions and may prevent misunderstandings as can arise when subsuming different conditions under the same concept. It also acknowledges the limitations of human robustness, and, at the same time, gives room to see the cured, the healed, and the habilitated as persons who can potentially change and improve from their former sound conditions.

## Recovery and the model of the triad: from what can we recover?

Recovery is generally understood as a process that involves a state of malady from which one 'comes back.' The biomedical practice is organized as if the case of injury is the ultimate understanding of recovery, what biomedicine considers to be a result of a successful treatment. However, like the state of disease, injury can overlap with illness and sickness, thus presenting a more complicated picture of recovery, which is not only an outcome of biomedical treatment.

Consider the following example: Maya injured her hand while working as a sous chef in a restaurant; she went to the hospital, and the nurse who welcomed her stitched her hand. After two weeks, the stitches melted into her skin, and Maya came back to work. We would normally say that Maya has recovered from her injury. Now consider another case: Sara is a professional swimmer who has been chosen to represent her country in the Olympic games for the first time in her career. Three days before the Olympic games begins, Sara was at home, making dinner and trying to relax. In a moment of distraction when cutting vegetables, she injured her hand badly. Like Maya, she went to the hospital and the nurse who welcomed her stitched her hand. Sara lost the momentum to break the world record, and the Olympic management in her country decided to send another swimmer instead. After two weeks, Sara's hand has recovered. Although the injury was physically minor, the circumstances left her completely troubled: Sara got depressed, could not return to a training routine, lost her prestigious scholarship, and felt that her life is meaningless. While one can argue that Maya has recovered when her hand returned to function, Sara, who went through the same biomedical procedure, did not recover; her recovery is not solely biomedical but also has phenomenological and social aspects.

**Fig. 1:**
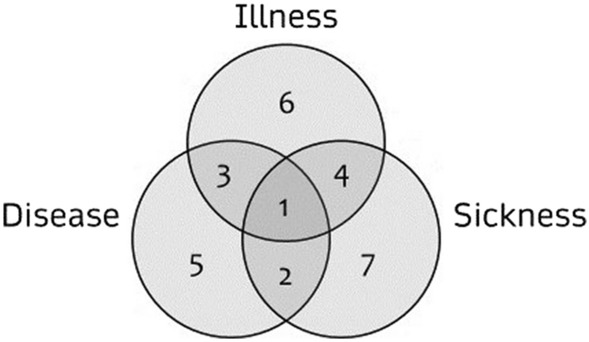
Twaddle’s model of the triad. The figure shows three partially overlapping circles. The left circle represents the state of disease, the central circle represents the state of illness, and the the right circle represents the state of sickness. Digit number 1 represents the area where the three circles overlap; digit 2 represents the area where disease and sickness overlap; digit 3 represents the area where disease and illness overlap; digit 4 represents the area where illness and sickness overlap; digit 5 represents the exclusive state of disease; digit 6 represents the exclusive state of illness; digit 7 represents the exclusive state of sickness (Hofmann 2002; Twaddle 1994)

As Sara’s case shows, when there are gaps between different perspectives on recovery, the use of the concept fails to capture the complexity of a given situation. In order to differentiate between these perspectives, I use the epistemic and normative understandings of disease, illness, and sickness suggested by the philosopher of medicine Bjørn Hofmann (2002), as well as the way that they are applied by Hofmann to the model of the triad (disease-illness-sickness) introduced by the sociologist of health Andrew Twaddle (1994) (Fig. 1).^3^ Hofmann's distinctions provide a conceptual framework from which it is possible to reveal the confusion that is often made between three different views of recovery, which I will show correspond to the ideas of disease, illness, and sickness. Hofmann defines *disease* as "a negative bodily occurrence as conceived of by the medical profession"^4^’^5^(Hofmann 2002). In contrast, he defines *illness* as "a negative bodily occurrence as conceived of by the person himself" (ibid.). According to Hofmann, *sickness* is "a negative bodily occurrence as conceived of by society and/or its institutions" (ibid.). Sickness can also be framed by either overt or covert norms that can result in stigmatization and discrimination (Hofmann 2016). In other words, he distinguishes between three agents that categorise the phenomenon of malady in three different ways: “disease” is pertain to medical and health care professions, “illness” to the first-person perspective, and “sickness” to social institutions that assign sickness to the individual. Accordingly, I will use the terms 'biomedical,' 'phenomenological,' and 'social' to describe each of these three distinct perspectives.

The biomedical perspective includes the views of both medical and healthcare professionals who decide whether someone is recovered from a disease or is still considered diseased. The phenomenological perspective is the first-person experience, the individual experience of being recovered or not. The social perspective is the view of the social environment on the individual as recovered or not. This view produces the expectations that society has of an individual. The social view includes laws and policies regarding eligibility for social benefits such as sick leave, as well as institutional and noninstitutional social behaviors, such as stigmatization and discrimination, and undesirable social attention such as staring at a disabled person or the expression of pity. Being eligible for sick leave or being a subject of these behaviors indicates that one is not recovered from a social point of view.

Applying these categories further, malady can be perceived from more than one perspective, so that two or three conditions can overlap (as in conditions 1,2,3,4 in the figure). Accordingly, a person can recover from disease (5), illness (6) or sickness (7), as well as two (2,3,4) or more of these conditions (1). Using Hofmann's definitions, injury is included under the broad category of disease as a condition under the medical and healthcare perspective.

While recovery may refer to improvement from all of these states, the concept does not differentiate between the different recovery paths. Following the model represented by Fig. 1, there are twelve cases which one can recover from as seen from one perspective but not from another. In the case a person is diseased, sick, and ill (condition 1 in the figure), he can recover from two perspectives and not from the third, or the other way around. Moreover, it is possible to become recovered from a biomedical and social perspective, in the sense of being viewed as healthy by the health system and by society, while still not feeling well from the first-person perspective. When one is considered recovered from one perspective (e.g., biomedical) but is not recovered from another perspective (e.g., first-person), one might confront difficulties caused by this discrepancy. Table 1 specifies the twelve different cases where such a discrepancy between the biomedical, phenomenological and social perspectives can occur. In the following, I will discuss and give examples that correspond to the cases in Table 1 of how one may recover in various ways as seen from the different perspectives mentioned, but still be ill, diseased or sick.

**Table 1:** specifies the twelve different cases where a gap between biomedical, phenomenological, and social perspectives can occur

Case number	Malady condition *t*1	"recovery" from … *t*2	Malady condition *t*3
1	Illness, sickness and disease [1]	Sickness and disease	Illness [6]
2	Illness, sickness and disease [1	Illness and sickness	Disese [5]
3	Illness, sickness and disease [1]	Illness and disease	Sickness [7]
4	Illness, sickness and disease [1]	Illness	Sickness and disease [2]
5	Illness, sickness and disease [1]	Disease	Illness and sickness [4]
6	Illness, sickness and disease [1]	Sickness	Illness and disease [3]
7	Sickness and disease [2]	Sickness	Disese [5]
8	Sickness and disease [2]	Disease	Sickness [7]
9	Illness and disease [3]	Disease	Illness [6]
10	Illness and disease [3]	Illness	Disese [5]
11	Illness and sickness [4]	Sickness	Illness [6]
12	Illness and sickness [4]	Illness	Sickness [7]

Condition *t*1 represents the malady condition before the “recovery” occurred (*t*2). Condition *t*3 represents the malady condition after “recovery” occurred (*t*2). The numbers in brackets refer to Fig. 1

It seems that recovery is more often associated with interventions made from the biomedical perspective, which are also assumed to include the recovery from sickness and illness. In case one in Table 1, patients who were diseased, ill and sick have recovered from the disease and the sickness, but not from the illness. As a result, the patients are not entitled to sick leave anymore, although they still feel ill. The recovery has happened only from the biomedical and the social perspectives, but not from the first-person perspective. Since they are biomedically well, they are expected to engage with everyday life as before. However, if they are still feeling ill, they might not perform in society as expected. In other words, there might be a lack of congruence between the expectations their environment has of them and the expectations they have of themselvess.

There are many cases in which people who have recovered from disease (and are not entitled to sick leave anymore) are still ill (see case one in Table 1). One example of this type of case (1) are patients who have myocardial infarction (MI) and suffer from disease, illness, and sickness and recover from the disease by medical intervention. MI treatment does not necessarily involve surgery or long cumbersome therapy; treatments via catheterization can be performed within half an hour, and at the end of it the patients are (more or less, see next section) biomedically recovered. This quick biomedical recovery makes the gap between the perspectives clearer. Research shows that many MI recoverers suffer from fatigue and feel depressed, stressed, or anxious, all of which negatively affects their quality of life and productivity, see for instance: (Mayou et al. 2000; Schaich et al. 2018). Thus, MI reveals a gap between recovery from the disease and recovery from an illness.^6^ The latter can be experienced as a life-threatening crisis that requires a different kind of treatment on a different time scale.

In case nine in Table 1, patients recovered only from the disease but not from the illness. Examples are patients who have recovered from bone sarcoma and are still facing psychosocial challenges. Research conducted at the Norwegian Radium Hospital and Oslo University Hospital has shown that two out of eighteen bone sarcoma patients are still struggling after recovery from the disease (Fauske et al. 2019). The participants stated that they lost interest in their hobbies, and, as a result, their social life was destroyed. They mentioned that they suffer from fatigue, lack of motivation, reduced cognitive function, and mental challenges (ibid.). The research emphasizes that "the late effects of cancer impacted their everyday lives to such an extent that they no longer consider lives to be meaningful" (ibid.).

Thus, recovery from a disease does not necessarily include recovery from an illness or provide the capacity to meet social expectations that come with recovery. When individuals become ill due to a medical condition (whether it is a disease, psychiatric disorder, injury, or disability), their world experience change. The phenomenologist S. Kay Toombs (1992) suggests five losses that characterize "a typical way of being" ill (ibid.)^7^: (1) *loss of wholeness*: since the illness draws awareness to the body, the individual can no longer take her own body for granted or as transparent; (2) *loss of certainty*: illness ruins the assumption of 'personal indestructibility' (ibid.) and forces the individual to admit vulnerability, which causes fear and anxiety; (3) *loss of control:* the individual is dependent on others both in decision making (on medical issues and others) and in completing tasks that previously did not require any assistance; (4) *loss of freedom:* the individual loses the freedom to act and make choices according to her system of values; and (5) loss of *familiarity*: the individual can no longer continue with her familiar everyday life as she did before in terms of activities and social relations (Toombs 1992).

The phenomenologist Havi Carel argues that an ill individual can also experience 'bodily doubt': "a radical modification of our bodily and other experience" (Carel 2016). Under this title, Carel lists three more types of loss: (1) *The loss of continuity* occurs when the illness interferes with the individual's everyday life flow. Things that were normal and did not require thought, now require effort and can change the individual’s personal goals (ibid.); (2) *The loss of transparency* occurs when the body is understood as a problem that requires attention and causes concerns (ibid.); (3) *The loss of faith in one's embodied existence* occurs when one loses the implied belief and certainty in one’s own body that allows one to perform everyday tasks (ibid.). In this sense, the term ‘recovery’ captures a desideratum of the ill to conceal the fragility of the body that the illness revealed.

Describing individuals as generally recovered when they are recovered from disease while still having an illness ignores the fact that patients need to recover from the implications of these losses, which can remain sources of suffering. Recovering from a disease does not mean that one immediately regains bodily confidence. It only makes sense that the experience of bodily fragility will not disappear at the same time as the disease. Furthermore, everyday life, including the social connections that have been affected by the illness, might take a longer time to recover from. In the opposite case, when individuals have recovered from being ill while still being diseased or disabled, our intuition is usually not to define them as recovered. From an 'insider perspective,^8^individuals can completely recover from being ill. Still, at the same time, from an 'outsider' view (a third-person perspective which includes biomedical and social perspectives), they will not be considered as such.

Cases number four and ten in Table 1 can refer to situations where individuals recover from an illness while still being diseased (10) or while being both diseased and sick (4). Both these cases can include diseases that are easily controlled by medications, like HIV and diabetes, as well as diseases that come in the form of seizures, like asthma or migraine. Between seizures, the individuals are still diseased but not ill. Case four also includes sickness. As mentioned above, sickness is not solely reflected by the recognition and support which society offers to the ill or diseased individual, but also reflects social costs that come with some conditions, like stigmas and discrimination. Patients with well-controlled HIV, who no longer feel ill, still carry the virus and, in many cases, the stigma.

The claim that one can be recovered from illness while being diseased is based on a stronger argument made by Carel, according to which one can be both ill and well at the same time (ibid.). Carel argues that some diseases can be well controlled (as in case four and ten), and others, like some disabilities (e.g., loss of vision), can be subject to adaptation (ibid.). Moreover, illness involves some gains that can lead to well-being, including "personal or spiritual insight, moral maturity, edification, and emotional clarity, as well as an ability to focus on what is most important and let go of other things"(ibid.). Thus, ignoring the process of recovery from an insider perspective is one way to neglect paying attention to the patients’ achievements in gaining well-being, even though they are still diseased.

One exception to the general preference of the biomedical view to define recovery is evident in psychiatry. 'The recovery model' in psychiatry acknowledges recovery, even when the symptoms are persistent (Jacob 2015). According to this approach, psychiatric recovery indicates "staying in control of one's life rather than an elusive state of return to the premorbid level of functioning" (ibid.). The model “emphasizes resilience and control over problems and life” (ibid.), so that one can live with a psychiatric disorder and be considered recovered at the same time.

However, the fact that the psychiatric recovery model emphasizes recovery from a first-person perspective and includes it in the biomedical perspective on recovery does not deny the potential to recover from the medical condition—even though recovery might be rare and hard to measure. In addition, according to the psychiatric model, it is not clear how recovery affects sickness. What are the social expectations, duties, and rights which psychiatric recovery entails, and how do they differ from the cases of those individuals who did not recover? Here again, the general idea of recovery leaves us with a vague understanding of the situation it is referring to.

In cases number three and five, we see that individuals may also be sick when they are no longer diseased (5) and ill (3). People who have recovered from illness and disease and feel well can still receive attention from society regarding their disease (as in the case of physical disabilities resulting from a disease). This attention can be positive, such as when one is eligible for disability benefits, or negative, as in cases where the disability is visible and triggers people to stare at the disabled person or even react in disgust or discriminating ways. In case number five, individuals are still struggling with both sickness and illness. People who have recovered from leprosy disease but still struggle with illness and sickness due to their physical appearance and discrimination are examples of such a case (van Haaren et al. 2016).

In other cases, individuals may be considered sick, even though there has never been a medical diagnosis (12) or they are no longer diseased (8). Hofmann mentions some examples such as chronic Lyme disease, whiplash, candida, and irritable bowel syndrome (IBS) where individuals can feel ill, and society (in many countries) entitles them “the status of being sick, but where the medical profession is not always able to identify or detect disease or injury" (Hofmann 2016). Hypothetically, individuals can have one of these conditions and then no longer feel ill while still being eligible for the rest of their sick leave (12).

One more problematic example of recovery from disease but not from sickness is having an asymptomatic case of COVID-19. Some countries follow advice which was previously given by the World Health Organization (WHO), according to which one must present two negative virus tests before being released from isolation (World-Health-Organization 17 June 2020). This advice was given to avoid the implications of false-negative results. In the time period between two negative results, people are then still tagged as sick and must be socially isolated, although they are technically virus-free. When there is a lack of laboratory supply or limited human resources to conduct the tests quickly, it may negatively affect isolated individuals.

Cases of sickness after recovery are rare in comparison to cases in which people are not entitled to sick leave, although they are ill (1, 11), diseased (2, 7) or both (6). In case eleven, the individuals feel ill and take sick leave. After a few days, they are not entitled to sick leave anymore. This situation is common in cases where the disease is not detected, whether it is a case without a familiar diagnosis or a mistake that leads to no diagnosis, which can be extremely harmful and even fatal for the individual. In some situations, the diagnosis is missed by the healthcare professionals because of a downgrading of the credibility of the ill person’s testimonial or due to testimonial exclusion that may lead to epistemic injustice^9^(Carel and Kidd 2014).

Until recently, individuals who had disorders like endometriosis, chronic fatigue syndrome, or fibromyalgia—which were not familiar to medicine— were ill but not recognized as having any biomedical problem and were therefore not considered sick. Their testimonial credibility about their illness was low, and they were rather tagged as lazy (Martínez-Quiñones et al. 2020; Nojima 2020; Wright 2018). Case number six is similar to case number eleven: the individuals are ill and diseased but not entitled to sick leave. This case is more common in places where there is no national requirement to offer paid sick leave, as for example in the United States. Under such circumstances, the illness and the disease conditions may worsen since the ill and diseased individuals are neither allowed sufficient time to rest nor are they offered treatment so that they can biomedically and personally recover.

Also in cases number two and seven, there is a medical condition, but here again the individuals are not considered sick. In these cases, the disease is no longer visible, and the patients feel well (as in case two, which initially included illness). Therefore, the patients are considered to be recovered and no longer sick. An example of a case where an individual are no longer sick (or no longer sick and ill) but still diseased would be one in which someone has a tumor that is regarded as removed but was not fully removed or which was considered by mistake as non-invasive. This is an actual medical condition that requires treatment, but it is not recognized by the treating professionals. In this case, the problem is not caused by different perspectives but by a gap in interpreting the situation within the biomedical sphere. Therefore, this case lies outside the focus of this paper.

We can then conclude that recovery is a general concept that does not indicate how one recovers. Additionally, the concept is often used in too narrow a sense, i.e., by considering only biomedical recovery while excluding the other perspectives. No less importantly, it does not give us information about how one did not recover. Furthermore, recovery does not indicate who declares this state, i.e., thanks to whom the person is no longer recognized as having a malady, and how it affects the other perspectives. The gaps between these perspectives can cause a reverse effect on the patients: the inability to recognize their illness or sickness (when recovered from symptoms), on the one hand, and their achievements to get well (when still having a disease), on the other hand, might have a negative influence on their condition.

## Re-covery? A comparison between soundness states before and after a malady

The psychiatric model of recovery leads me to my second point in this conceptual analysis of recovery: its inherent expectation that the recovering agent *comes back* to the premorbid state before the malady occurred. That is to say, the primary sound state (S*T1*) is equivalent to the new sound state (S*T3*) after a malady (*T*1–*T*3). I argue, however, that in most of the cases, as we saw in the case of psychiatry, recovery *does not indicate coming back to a former state* but describes different sorts of relations between S*T1* and S*T3.*

My view on recovery as a process that goes forward echoes the vital perspective in philosophy of life science, according to which health and disease are understood as a function of new biological norms in an individual organism and its interaction with its environment. The German neurologist and psychiatrist Kurt Goldstein wrote the following from his holistic perspective about well-being in a stage of incapability:

"*[B]eing well* means to be capable of ordered behavior which may prevail despite the impossibility of certain performances which were formally possible. But the new state of health is not the same as the old one[…] to become well again, in spite of defects, always involves a certain loss in the essential nature of the organism. This coincides with the reappearance of the order. A *new individual norm* corresponds to this rehabilitation" (Goldstein 1939).

Thus, according to Goldstein, although the state of health has changed, one can gain well-being by accruing new norms. Having been deply influenced by Goldstein's writing, George Canguilhem, a French philosopher and physician, wrote: "[N]o cure is a return to biological innocence. To be cured is to be given new norms of life, sometimes superior to the old ones. There is an irreversibility of biological normativity"(Canguilhem 1989). In other words, according to both Goldstein and Canguilhem, there is no “re-covery” in the sense that the new stage is the same as the premorbid stage.

One can reject Goldstein and Canguilhem's view by defining the direction of "re-covery" as a movement between binary states: from an unhealthy state back to a healthy state, or from an unrobust state to a robust state, or as a return from unwellness to wellness. Accordingly, one can be healthy, robust, and well—or not, so there are no degrees of health, robustness, and well-being. Moreover, on this view, recovery is to be understood as an event and not as a process.^10^ Understanding recovery as an event contradicts received knowledge about the biological process that happens when the body recovers, as well as many phenomenological experiences of recovering, see, for example: (Deegan 1988; Groven and Dahl-Michelsen 2019). Thus, recovery should not be perceived as a marker for switching between binary states.

As a process that incorporates various phases of soundness and malady, recovery should thus be gained when S*T3* = S*T1*. De facto, the concept of recovery is also used when the new sound state of a patient is better (S*T3* > S*T1)* or worse (S*T3* < S*T1)* from his state before the malady occurred. In addition, it is used in situations where one cannot evaluate the relation between the two stages (S*T3*?S*T1*), i.e. in cases that, in principle, have required the suspension of judgment. On this view, the process of recovery creates a new condition of soundness that does not have to be the same as the former one.

Let us have a look at some examples of the four possible relations between S*T1* and S*T3*. From the biological perspective, in some cases, one can detect an *improvement from ST1 to ST3*. Some diseases can be considered low-dose stressors that improve the body's resilience, especially in early life stages (Li et al. 2019). For example, this happens in the immune system through the production of antibodies, which reduce the probability of the reoccurrence of the same disease, so the process of recovery makes the body more immune than before.^11^ Recovery from an illness can also result in an improvement of well-being, which affects the individual’s state of health. In the literature, this phenomenon is referred to as «Posttraumatic Growth», which includes the experience of meaningful family relationships, the experience of meaningful engagement, and appreciation of life (Tolleson and Zeligman 2019; Zhai et al. 2019). Recovery from sickness can result in a more productive state, for example when people realize that they are not happy with their job during sick leave and decide to do something else, which they like, and therefore invest more in work than before. When using the concept of recovery, one ignores the potential of improvement of the sound condition. The idea of recovery limits the vital power of life to evolve and become more immune, robust, and productive.

On the other hand, recovery is also used in situations where one's *condition becomes less evident compared to the former state*. From the biomedical perspective, many mechanical traumas can be considered recovered, although they cause a minor deficiency in bones and tissues. Even the case of a minor cut in the skin can leave one with a scar. This is an inadequacy that is not necessarily harmful either to the individual or to his or her society.

One can also think about the degree of soundness as a sum of the three perspectives together. Then, in some conditions, we could detect a decrease in soundness that can be harmful to individuals' lives. The case of the perception of illness in leprosy-cured individuals who experience shame and stigma is an example for such a condition (van Haaren et al. 2016). In addition, underestimating one of the conditions can result in a lower state of soundness from a long-term perspective. Leprosy-cured individuals who suffer from stigma could become more ill than when they were diseased, thereby becoming more vulnerable to develop psychosomatic diseases.

Another example is cancer-free individuals who experience 'extreme persistent fatigue' that affects their social performance even years after recovery (Rosman 2009). Such a condition is not necessarily recognized as sickness and may put stress on cancer-free individuals, which might affect their medical situation. Thus, a discrepancy between recovery from disease and recovery from illness can affect the expectations that society has of “recovered” individuals and may also result in worsening illness conditions and potentially cause more diseases and sickness.

In some situations, it is *unknown whether the malady has resulted in a better or worse soundness condition*. A current example is the state of recovering from COVID-19. According to the World Health Organization (WHO), "there is currently no evidence that people who have recovered from COVID-19 and have antibodies are protected from a second infection" (World-Health-Organization 24 April 2020). The same is true in situations where a virus remains latent and has the potential to reactivate itself, either in the same or a different form. Varicella-zoster virus is an example of such a case (Gershon 2017).

Another example in which it is hard to determine whether one gains the same state of soundness is when illnesses, and especially severe illnesses, change ‘the structure of experience’ (Carel 2016). People can feel ambiguity concerning their illness, struggle with it, and experience personal growth simultaneously (Kalitzkus and Matthiessen 2010). Experience is not subject to accurate quantitive measurement, and different structures of experience cannot easily be associated with a better or worse condition.

From a social perspective, it is sometimes hard to know whether someone is less or more socially active or productive after a malady than before, since the malady can change one's life path and affect one’s social and work environment. Individuals can have fewer social connections but more meaningful interactions than before or work in different professions in which productivity is measured differently. Thus, although there is no way to make a judgment about the new state of soundness, the concept of recovery is still used in relation to the process.

*When, then, has one actually re-covered?* Biomedical markers and conditions for which deviations from the statistical norm are associated with a disease are both examples where one can regain the same condition as before the abnormality occurred, e.g., in conditions of high blood pressure and high sugar levels. Recovered patients can also express that they feel the same way as before they became ill. More common is thinking about recovery in terms of sickness, where an individual comes back to perform in society to the same degree as before. An important social measurement in this respect is the ability to work the same number of hours at the same efficiency rate as before the malady.

Measuring soundness across time is a great challenge. This measurement should take into account many different variables—biomarkers, the nature of one’s relationship with family and friends, and the amount of sick leave days used, to name a few—and combine various methodologies, e.g., clinical assessment, blood tests, questionnaires, experiments on stigmas related to specific diseases, and so on. Soundness also includes many other aspects of individuals' lives that need to be tracked to obtain a full picture of an individual’s state of soundness as a whole. In recent years, new initiatives such as P4 medicine^12^has increased the need to form accurate tools to measure soundness. That is to say, such precise tools are not yet available.

## A conceptual restart and new terminology

To describe various paths towards soundness more accurately, I propose a conceptual restart: instead of understanding the process away from malady as going back from the malady to the former sound state, I suggest viewing the process as starting from the malady and moving forward towards the new sound state. Changing the focus in this way enables us to bypass the hard and, in many cases, nonproductive task of measuring soundness.

Moving from the malady forward to the new sound state can be achieved from the three different perspectives previously discussed: biomedical (disease, injury), first-person (illness), and social (sickness). Correspondingly, I suggest three types of movements from a malady to soundness: curing, healing, and habilitating. The new sound states can have different qualities that are not easily measured quantitatively. Although these three concepts refer to different understandings of movement away from maladies of everyday life, using them in a constricted sense with regard to only one of the perspectives can be useful for tracking a patient's development more accurately. Moreover, having different terms to describe these different perspectives allows for an awareness of potentially problematic gaps between them. In other words, my suggestions aim to make the differences between these perspectives more visible and avoid the harm that can be caused by conflating them when one uses a general and common terminology.

As suggested by Canguilhem (Canguilhem 1989), being cured refers to the growth of new biological norms, without tying the new condition to the former one. Using the term «cure» regarding a specific medical condition allows for patients to be tracked more effectively with respect to their medical conditions. The definition of cure is subject to medical and healthcare perspectives, and, as such, whether someone is cured can be measured medically, using methods like scans and laboratory biomarkers.

Curing should also be specified according to different aspects of the biomedical view. Diseased individuals receive different kinds of attention and care from the healthcare system. In some cases, there is no treatment and no way to reduce one's suffering since little is known about the condition. Sometimes being in a risk group requires biomedical intervention and medical tracking, as in the case of high blood pressure, non-active HIV, or a high risk of breast cancer. However, other risk groups do not receive much attention from health providers until the risk becomes a reality, as in the case of varicella-zoster virus that remains in the body after recovery from chickenpox and that can be activated later as shingles disease (Gershon 2017).

In addition, in some cases individuals are considered cured, although they still possess other medical issues considered as side effects or caused by either the medical condition itself or medical treatment. There could be short- or long-term effects; some appear immediately and others late in life. One example of this may be the long-term cardiovascular and respiratory effects of COVID-19 on people who are considered cured, since they are no longer carrying the virus (World-Health-Organization 9 September 2020).

Thus, it is crucial to be accurate regarding the condition of which one has been cured. It is no less important to notice the conditions of which one has not been cured (as in the case of biomedical side effects), whether these conditions are treatable or not treatable. Further, it is important to note what are the known biomedical risks that the new condition bears. Cure is not a binary concept, insofar as a person can be cured of one medical condition, but not from another.

The etymology of the verb «to heal» originates from old English, meaning "to make whole" (Online-Etymology-Dictionary n.d.). What does it mean to be whole? Put differently, when is one not whole, or when does one change to be a different whole? According to the phenomenologist Fredrik Svenaeus (2019, 2000), illness is the experience of uncanniness or 'unhomelike being in the world' that arises from changed conditions of embodiment. Carel describes the experience of illness as ‘the shrinking of one's world’ (Carel 2014, 2016). In light of the phenomenological view, I suggest that healing is a process of creating a new feeling of homelikeness, which makes one whole with one’s body, expanding one’s world to the degree that allows for wellness.

This sense of wholeness is understood on the basis of a first-person experience (sometimes it can also be related to religious or spiritual experiences, see: (Boyd 2000). Thus, being healed is a condition that one can only declare about oneself. To learn about how one experiences oneself, one needs to be attentive to phenomenological experiences that can be expressed through both written and spoken language (for example, through conversations and questionnaires) and other methods of expressions, such as the act of making art. In a clinical context, this means being attentive not only to the medical history of the disease but also to ‘the clinical narrative’, i.e. the patient’s experience of being ill (Toombs 2001).

These strategies of being attentive are crucial for both tracking the healing process and supporting the process by creating empathy. According to Toombs (ibid.), to be empathic to the ill person is to see the world through his or her eyes and relate to the unique way they experience the illness (ibid.). Empathy can create common ground to understand what is needed to support the patient in the healing process. The way illness is experienced can influence the curing process and vice versa, but they are not always synchronized. The healing process can also be affected by how society perceives the ill, by contributing to either enhancing or harming an image of oneself as a valuable and capable individual.

Habilitation is a condition in which individuals who have disabilities (from birth or acquired through life) have gained or regained the ability to function in society. Habilitation is subject to social views, and it is formed through social institutions and healthcare policymakers. Social ability can be measured through various prisms such as productivity at work and the ability of a person to interact with other people and create social connections. Another aspect of habilitation is related to one’s entitlement to sick leave. The definition of habilitation changes regarding the formation of policies in each society or social organization.

Habilitation can also be acquired by adaptation or habituation to a new condition. The ability to adapt and the degree of adaptation can be affected by society and the surrounding material conditions. Empathy also plays an important role in one's adaptation, as it allows us to include others in society. Empathy motivates people to create technological advancements that help disabled people accomplish tasks which they couldn't accomplish before because of their disability. Empathy can also affect the inclusive design of the public sphere that can support the habilitation process. Similarly, the lack of empathy, discrimination, and stigmas all impair the habilitation process.

Curing, healing, and habilitating are three different processes that can mutually affect each other under some conditions. Although there are possible links between these conditions, they are not always synchronized. Sometimes one of the processes is not possible, such as in the case of curing a chronic disease. In other cases, they take an unequal time or require different kinds of treatment or resources. For achieving the best sound condition possible in a given situation, it is necessary to consider all three processes.

## Concluding remarks

In this paper, I have shown that recovery is an inadequate concept as it glosses over important distinctions and even misrepresents the process of moving away from malady as "going back." I argued that the concept of recovery neglects important nuances needed to distinguish between the biomedical, first-person, and social perspectives. I also claimed that there are many circumstances where we make use of the concept of recovery, although the person in question has not regained the same degree of soundness. I therefore conclude that referring to recovery is only accurate in specific situations, when one is coming back to the same state of soundness as one had before the malady occurred, and it is a relevant subject of discussion whether this is ever the case. In all other cases, it is a misleading concept. To gain a more adequate understanding, I have proposed re-thinking the direction of the process in question. I define the process of moving away from malady as a move forward towards a new state of soundness. By pointing out a different direction of the process, I disconnect the new sound state and the premorbid state, arguing that the need to see them in light of each other is redundant. I have suggested three terms to describe this movement forward: 'curing' (biomedical perspective), 'healing' (first-person perspective), and 'habilitation' (social perspective).

There are two related concerns that could come to mind at this point. First, abandoning the concept of recovery altogether might not be seen as a realistic expectation. Second, in addition to the specific processes of curing, helaing, and habilitation, there are still situations in which one would like to refer to all of them together, as they can still be interpreted as belonging to the same kind. To dispel these concerns, I suggest another small but meaningful change: replacing the term ‘recovery’ with the term ‘recovery processes’. The word ‘process’ has a connotation of progress, which redirects the prefix ‘re’ forwards. Using the plural form is a reminder of the different ways in which one can recover. This new terminology allows for a better understanding of the complexity of malady and soundness states and points to the importance of being attentive to the differences between them and their possible reciprocal effects.

## Footnotes

1 I employ the term «soundness» to refer to all these states at once.

2 I use «malady» as a common term referring to all these negative states at once.

3 This figure stems from Hofmann’s (2002), which refers to Twaddle’s original (1994).

4 I take psychiatric disorders to be included under the title of disease (with possible overlaps with illness and sickness) although Hofmann and Twaddle’s models include only somatic diseases, see: (Hofmann 2002). The reason for this is that my interest lies in psychiatry as part of the biomedical perspective, and not in the question of whether mental disorders are diseases. I follow the distinction made by Kendell (2001) between mental illness and psychiatric disorders and the view that the latter is the subject of contemporary medicine.

5 Occurrence, here, according to Hofmann, means a process, state, or event (Hofmann 2002).

6 Interestingly, one can also feel ill when becoming part of a risk group, without actually having the disease. MI and COVID-19 are examples of cases that being in a risk group can cause high anxiety and change of lifestyle that can result in a negative psychological impact on the individual. Avoiding physical activity in the case of MI risk or self-isolation in case of COVID-19 risk can have a great effect on the individual, no less than in case of having the disease.

7 Carel refines Toombs’s account by arguing that these losses can be experienced differently whether the condition is temporary, permanent, or progressive, and they can also vary across cultures (Carel 2016).

8 For more on the distinction between ‘insider’ and ‘outsider’ perspective see: (Carel 2016).

9 Carel and Kidd analyse testimonial and hermeneutical injustice in healthcare as two forms of epistemic injustice in the sense articulated by the philosopher Miranda Fricker (2007).

10 It is also important to note that the process of recovery does not have to be linear, and that one can move between better and worse conditions several times and to different degrees while recovering.

11 In some cases, immunity can also be regarded as a deficiency, for example if it reduces the presence of pre-existing antibodies for other diseases, as recent studies have shown regarding measles disease, c.f: (Mina et al. 2019).

12 P4 stands for: Predictive, Preventive, Personalized, and Participatory Medicine. P4 is a holistic approach that requires the collection and analysis of vast amounts of health-related data on individual patients. While in the past medicine focused on the treatment and prevention of diseases, P4 medicine emphasizes the medicalization of health and well-being (Clarke et al. 2003; Vogt et al. 2016).
